# Complete chloroplast genome of *Isoetes orientalis* (Isoetaceae), an endangered quillwort from China

**DOI:** 10.1080/23802359.2023.2183070

**Published:** 2023-02-28

**Authors:** Yanqing Li, Xi Chen, Xiaoyan Lin, Yufeng Gu, Baodong Liu, Rongjing Zhang

**Affiliations:** a College of Life Sciences, South China Agricultural University, Guangzhou, China; b Key Laboratory of National Forestry and Grassland Administration for Orchid Conservation and Utilization, The National Orchid Conservation & Research Center of Shenzhen, Shenzhen, China; c Life Science and Technology College, Harbin Normal University, Key Laboratory of Plant Biology in Colleges of Heilongjiang Province, Harbin, China

**Keywords:** *Isoetes orientalis*, hexaploid, Isoetaceae, phylogeny, plastome

## Abstract

*Isoetes orientalis* is an endangered hexaploidy species of Isoetaceae in China and the complete chloroplast genome of this species has not been reported. In the present study, a complete chloroplast genome of *Isoetes orientalis* (Isoetaceae) was assembled and annotated. This chloroplast genome has a circular structure of 145,504 bp in length, comprising a pair of inverted repeat (IR) regions of 13,207 bp each, a large single-copy (LSC) region of 91,864 bp, and a small single-copy (SSC) region of 27,226 bp. The chloroplast genome contains 136 genes, including 84 protein-coding genes, 37 tRNA genes, and eight rRNA genes. Phylogenetic analysis showed that *I. orientalis* was closely related to *I. sinensis.* These results provide additional resources for future studies on *Isoetes* from China and across the globe.

## Introduction

**Figure 1.**
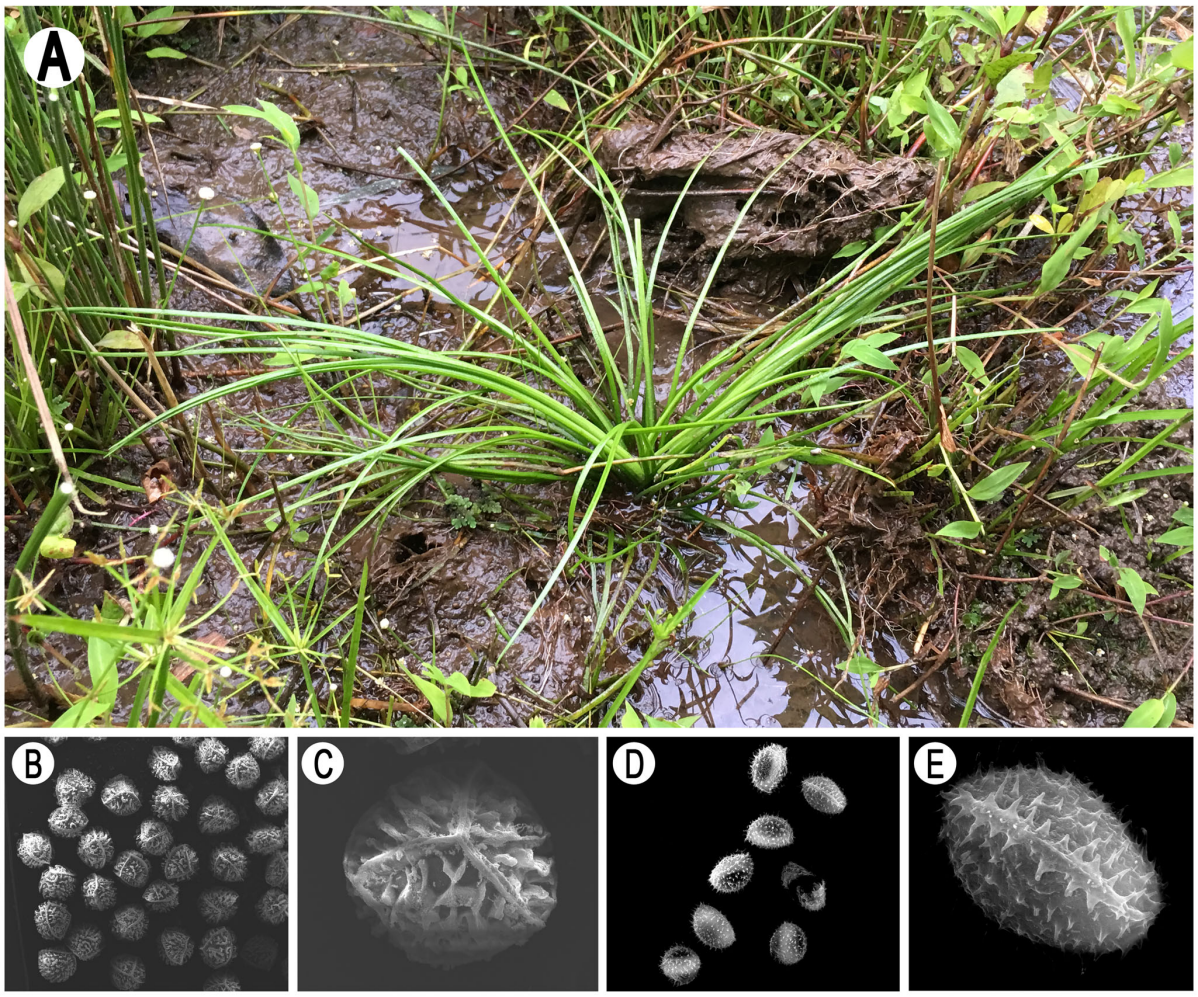
*Isoetes orientalis* (Isoetaceae). (A) Habitats; (B, C) megaspore; (D, E) microspore.

*Isoetes orientalis* Hong Liu & Q. F. Wang, an endangered hexaploid quillwort belonging to Isoetaceae, was reported in 2005 (Liu et al. 2005) and previously identified as *I. sinensis* (Pang et al. 2003; Ye and Li 2003; Chen et al. 2004; Kang et al. 2005), because this species has 66 chromosomes and spore features are different from *I. sinensis*, it was identified as new species. *Isoetes orientalis* is an emergent aquatic plant growing in the marshes of Songyang County of Zhejiang Province, China (Liu et al. 2005). Megaspores of this species have cristate-reticulate ornamentation, while microspores have echinate-tuberculate ornamentation (Liu et al. 2005, 2008) (Figure 1(B–E)).

Using NGS to infer evolutionary relationships with genomic-scale datasets allows researchers to resolve the branching order within rapid radiations and obtain a more robust phylogenetic framework (Wei et al. 2017; Wei and Zhang 2020). Plastid genome (plastome) DNA sequences have been extensively used in recent plant molecular systematics because of their mode of uniparental inheritance and high content of informative loci (Givnish et al. 2010; Jansen et al. 2011; Ruhfel et al. 2014; Givnish et al. 2015; Lu et al. 2015; Ross et al. 2016; Schafran et al. 2018). As an endangered species, *Isoetes orientalis* is also the only hexaploidy species in China, which would be very important in the speciation path of Isoetaceae of China even East Asia. Therefore, it will be significant to know its complete chloroplast genome sequence.

## Materials and methods

In the present study, fresh leaf material was obtained from the collection sites of Andaihou Village, Songyang County, Lishui City, China (119.273422 E, 28.271808 N) (Figure 1) and dried with silica. Specimens (voucher no.: Yufeng Gu Fern08748) were deposited at the Herbarium of the National Orchid Conservation Center (NOCC). Silica-dried material was sent to Shanghai Majorbio Bio-pharm Technology Co., Ltd. (Shanghai, China) for DNA extraction and sequencing, performed on an Illumina HiSeq X Ten platform (Illumina, San Diego, CA). The plastid genome was assembled using GetOrganelle v1.7.5 (Jin et al. 2018) using default parameters, and the results viewed and edited by Bandage v0.8.1 (Wick et al. 2015). The assembled chloroplast genome was annotated by Geneious Prime 2021.0.3 (https://www.geneious.com) (Kearse et al. 2012) with *I. nuttallii* as a reference at 90% similarity.

**Figure 2.**
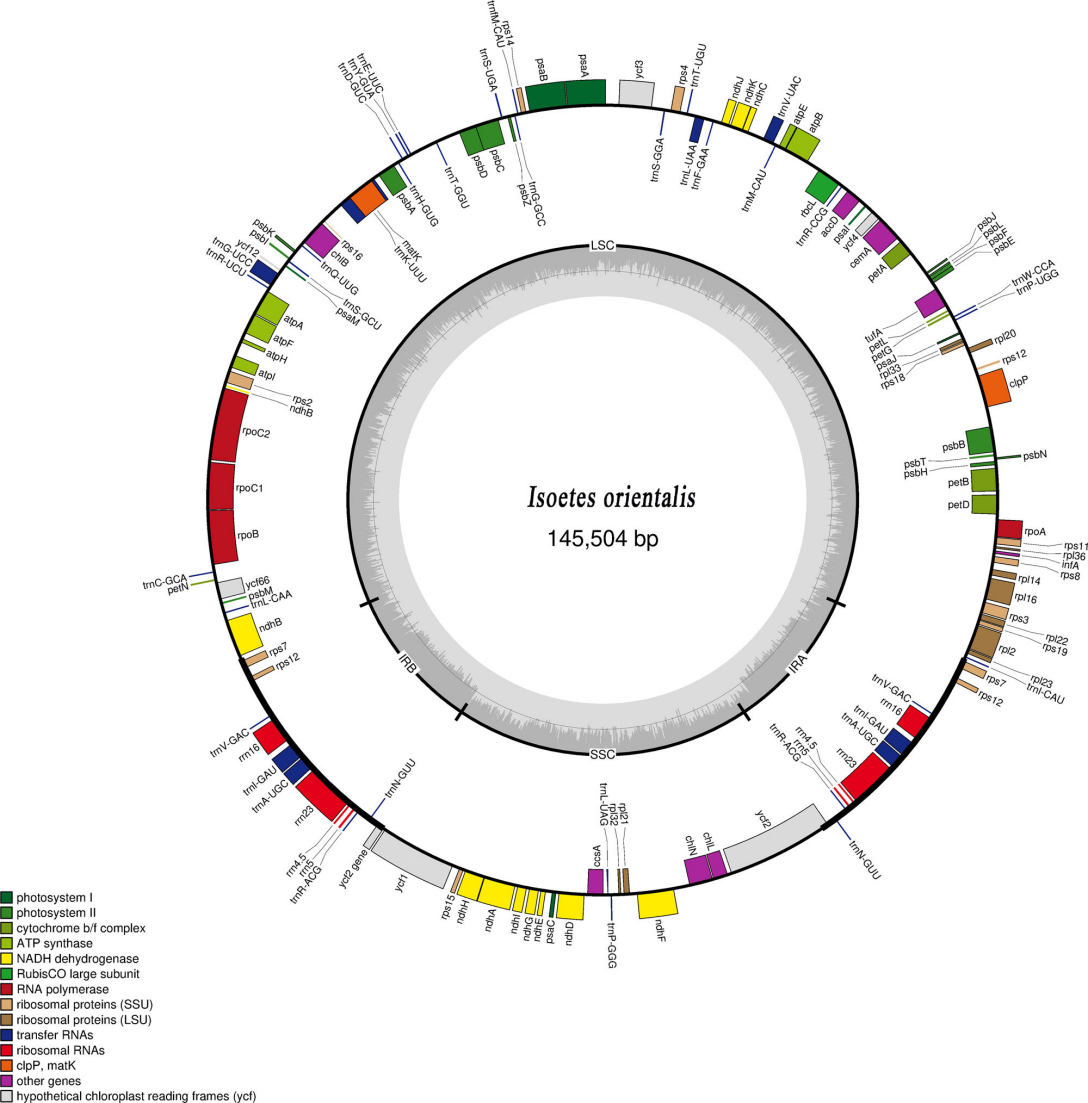
Chloroplast complete genome map of *Isoetes orientalis* (Isoetaceae).

We drew the chloroplast complete genome map of this quillwort species (Figure 2) in OGDRAW – Draw Organelle Genome Maps (https://chlorobox.mpimp-golm.mpg.de/OGDraw.html). To find the phylogenetic position of *I. orientalis*, molecular phylogenetic analysis was carried out with 15 published, complete chloroplast genomes of *Isoetes* downloaded from GenBank.

Coding sequences (CDS) were extracted from the annotated sequences, then they were aligned using mauveAligner in Geneious Prime 2021.0.3. By employing a progressive algorithm and assuming collinearity, poorly aligned regions were excluded from the complete plastome dataset using Gblocks v0.91b in PhyloSuite v1.2.2 (Zhang et al. 2020). Using nucleotide as the type of sequence, up to half gap positions were allowed, and other parameters were set as default settings. For phylogenomic analysis, the resulting alignment was subjected to ML analyses performed using IQ-TREE v. 1.6.12 (Lam-Tung et al. 2015) with 10,000 bootstrap replicates. The best-fitting model was selected by ModelFinder (Kalyaanamoorthy et al. 2017) and implemented in IQ-TREE.

## Results

Complete plastid genome sequence of *I. orientalis* (GenBank accession: OL467336) was 145,504 bp in length containing a large single-copy (LSC) region of 91,864 bp, a small single-copy (SSC) region of 27,226 bp, and a pair of inverted repeats (IRs) of 13,207 bp each. A total of 136 genes were annotated, including 84 protein-coding genes, 37 transfer RNA (tRNA) genes, eight ribosomal RNA (rRNA) genes, and seven genes, *infA*, *tufA*, *accD*, *rps2*, rps*16*, a copy of *ndhB* and *ycf2*, are free in function. The overall GC content was 38.0%.

The complete plastid genome sequence of *Isoetes orientalis* (Isoetaceae) is 145,504 bp in length, comprising a pair of IR regions of 13,207 bp each, an LSC region of 91,864 bp, and an SSC region of 27,226 bp. The chloroplast genome contains 136 genes, including 84 protein-coding genes, 37 tRNA genes, and eight rRNA genes. The overall GC content is 38.0%.

**Figure 3.**
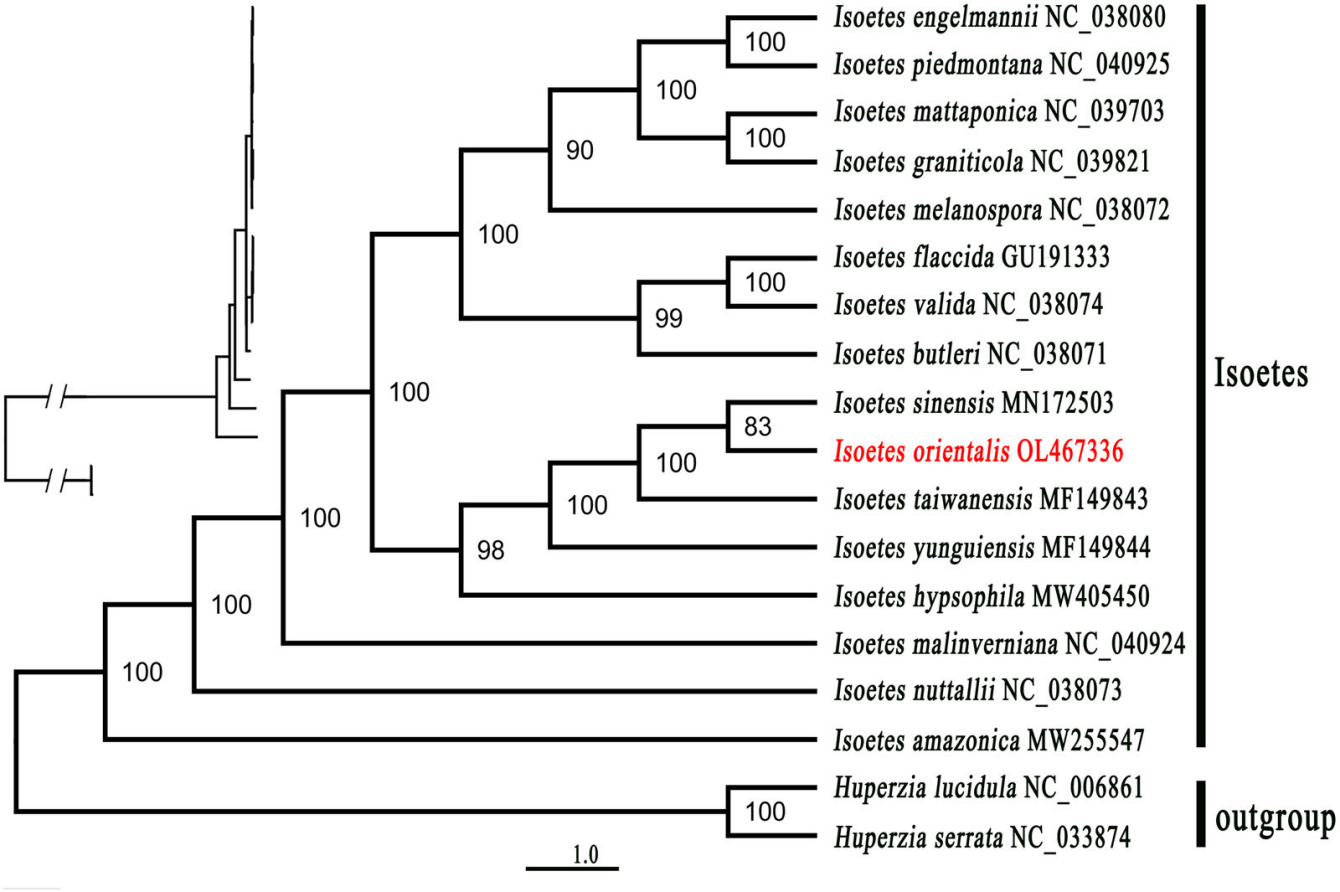
Maximum-likelihood phylogenetic tree of 16 *Isoetes* species based on CDS sequences by IQ-TREE, two *Huperzia* was set as outgroup. The number on each node indicates the bootstrap support value.

ML (maximum-likelihood) tree (Figure 3) indicated that *Isoetes amazonica*, *I. nuttallii*, and *I. malinverniana* were found as three clades solely. *Isoetes orientalis* was clustered with *I. sinensis* with support ratio 83%, while *I. taiwanensis* showed a sister relationship with *I. sinensis* and *I. orientalis* with 100% bootstrap support values. *I. yunguienesis* was a sister to the clade formed with the above three species *I. taiwanensis*, *I. sinensis*, and *I. orientalis*. All the above four species formed a sister group with *I. hypsophila*. All the five species collected from China formed sister clade with *I. engelmannii*, *I. piedmontana*, *I. mattaponica*, *I. graniticola*, *I. melanospora*, *I. flaccida*, *I. valida*, and *I. butleri.*

## Discussion

Study on the evolution of Isoetaceae using complete chloroplast genomes has been carried recently (Pereira et al. 2021), but only a few species were employed. There are nine species of *Isoetes* reported in China, but only four of which were published chloroplast complete genomes. When to take further study on the Isoetaceae of China, more chloroplast complete genomes will be needed in the future. The results of the present study will supplement the chloroplast genome data of *Isoetes* collected in China and hold great significance in the study of this genus.

## Supplementary Material

Supplemental Material

## Data Availability

Genome sequence data that support the findings of this study can be obtained from GenBank of NCBI (https://www.ncbi.nlm.nih.gov/) under the accession no. OL467336. Associated accession numbers are listed as BioProject PRJNA781054, SRA SRS11088792, and Bio-sample SAMN23227414.
