# Supplementary material for: Complete chloroplast genome of Isoetes orientalis (Isoetaceae), an endangered quillwort from China
Source: Mitochondrial DNA B Resour. 2023 Feb 28;8(3):342–6. doi: 10.1080/23802359.2023.2183070 (PMC9980155; doi:10.1080/23802359.2023.2183070)
Supplement: Supplemental Material [file TMDN_A_2183070_SM7482.docx]

Supplement Materials

QC data of the sequencing result

Table S1 Data Output statistics

| Raw reads | Clean reads | Raw base (G) | Clean base (G) | Effective Rate(%) | Error Rate(%) | Q20(%) | Q30(%) | GC Content(%) |
| --- | --- | --- | --- | --- | --- | --- | --- | --- |
| 58,243,726 | 57,881,702 | 8.74 | 8.68 | 99.38 | 0.03 | 95.56 | 88.68 | 41.92 |


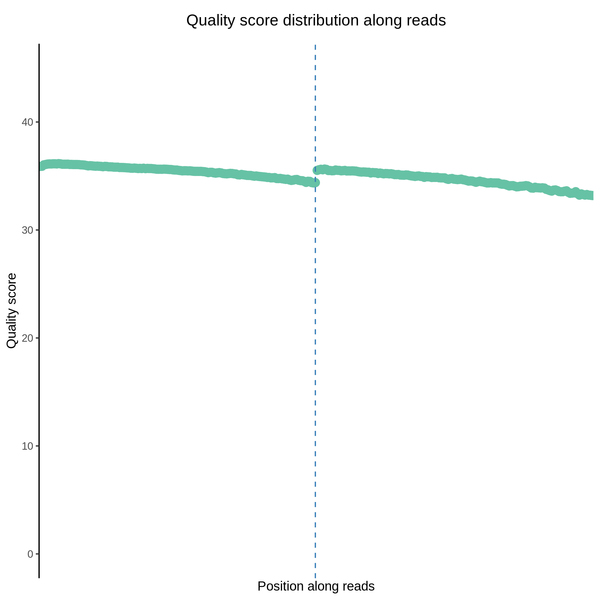


Figure S1 Data quality distribution


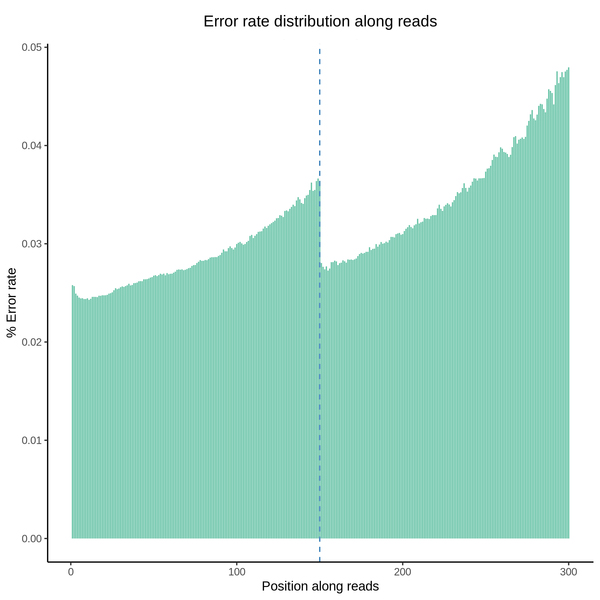


Figure S2 equencing error rate distribution map
